# Advancing Drug Discovery and Development from Active Constituents of Yinchenhao Tang, a Famous Traditional Chinese Medicine Formula

**DOI:** 10.1155/2013/257909

**Published:** 2013-09-26

**Authors:** Aihua Zhang, Hui Sun, Shi Qiu, Xijun Wang

**Affiliations:** National TCM Key Lab of Serum Pharmacochemistry, Key Lab of Chinmedomics and Key Pharmacometabolomics Platform of Chinese Medicines, Heilongjiang University of Chinese Medicine, Heping Road 24, Harbin 150040, China

## Abstract

Traditional Chinese medicine (TCM) formula has been playing a very important role in health protection and disease control for thousands of years. Guided by TCM syndrome theories, formula are designed to contain a combination of various kinds of crude drugs that, when combined, will achieve synergistic efficacy. However, the precise mechanism of synergistic action remains poorly understood. One example is a famous TCM formula Yinchenhao Tang (YCHT), whose efficacy in treating hepatic injury (HI) and Jaundice syndrome, has recently been well established as a case study. We also conducted a systematic analysis of synergistic effects of the principal compound using biochemistry, pharmacokinetics and systems biology, to explore the key molecular mechanisms. We had found that the three component (6,7-dimethylesculetin (D), geniposide (G), and rhein (R)) combination exerts a more robust synergistic effect than any one or two of the three individual compounds by hitting multiple targets. They can regulate molecular networks through activating both intrinsic and extrinsic pathways to synergistically cause intensified therapeutic effects. This paper provides an overview of the recent and potential developments of chemical fingerprinting coupled with systems biology advancing drug discovery towards more agile development of targeted combination therapies for the YCHT.

## 1. Introduction

Currently, the treatment of human diseases has seen a shift from the “1 disease, 1 target, 1 drug” and “1 drug fits all” modes to hunt for combination therapies [1]. It represents a triumph for modern medicine and provides fertile ground for modern drug discovery and development [2–5]. Interestingly, TCM is an essential part of the healthcare system in most Asian countries and has attracted attention even in European and North American countries. The therapeutic efficacy of TCM, usually attributed to the synergistic property of multiple herbs and constituents, has advocated combinatorial therapeutic strategies for 2,500 years using prescriptions called formulae that minimize adverse reactions or improve the therapeutic efficacy [6]. 

**Figure 1:**
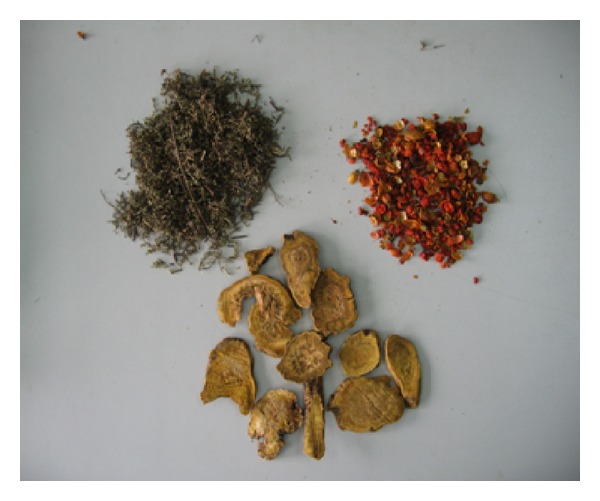
Prepared herbal medicines of Yinchenhao Tang.

Generally, the famous formulae include four elements: the monarch (which plays the most important role in the formulae), the minister (which increases the effectiveness of the monarch herb), the assistant (which helps the monarch and minister herbs reach their target positions), and the servant (which can reduce the adverse effects and/or increase the potency of the whole formulae). They work together harmoniously to achieve an ideal therapeutic effect [7]. However, their precise mechanisms are poorly understood and must be addressed using a molecular approach. Yinchenhao Tang (YCHT, Figure 1), a well known Chinese herbal formulae, was recorded in “*Shanghanlun*” which a classic resource on TCM written by Zhongjing Zhang (150–215 A.D.) and was officially listed in the Chinese Pharmacopoeia (China Pharmacopoeia Committee, 2010). It consists of *Artemisia annua* L. (the monarch herb), *Gardenia jasminoides* Ellis (the minister herb), and *Rheum Palmatum* L. (the assistant and servant herb) and has been used for more than a thousand years to treat jaundice and liver disorders. It possesses many effects, such as treating cholestasis, liver fibrosis, hepatitis C, biliary cirrhosis, and cholestatic liver diseases [8]. 

Synergism is a key principle of TCM [9], and understanding the synergistic effects of YCHT represents an even greater challenge than usual. Fortunately, its mode of function can be revealed using modern biochemical analyses. YCHT can be analyzed using chemical fingerprinting, pharmacokinetics, and systems biology approaches, promising new schemes and patterns of advancing drug development from active constituents of Chinese medical decoction. 

## 2. Pharmacological Study of YCHT

YCHT, also called Inchin-ko-to or TJ-135 in Japan, is known to inhibit hepatocyte apoptosis as well as promote the secretion and excretion of bile. Ogasawara et al. showed that the preoperative administration of YCHT can provide beneficial effects in promoting hepatic regeneration and preventing postoperative hepatic failure [10]. It may be an effective agent in postoperative management of liver resection by its potent choleretic effect. Alcohol liver disease (ALD) remains one of the most common causes of chronic liver disease in the world [11]; YCHT plays a certain role in its therapy. A study applied metabonomics to characterize the hepatotoxicity induced by alcohol and intervention of YCHT. The greatest difference in metabolic profiling was observed from alcohol treated rats when compared to the control and YCHT treated rats using a multivariate statistical analysis method such as the principal components analysis (PCA). Consequently, three potential biomarkers were detected and associated to alcohol hepatotoxicity. The trend lines revealed the presence of those biomarkers in alcohol treated rats when compared to control and YCHT treated rats as an obvious difference. It demonstrated that YCHT indicates some intervention on alcohol hepatotoxicity by improving perturbed metabolic process. 

To optimize the animal model of liver injury that can properly represent the pathological characteristics of dampness-heat jaundice syndrome of TCM [12]. The liver injury in the model rat was induced by alpha-naphthylisothiocyanate (ANIT) and carbon tetrachloride CCl_4_, respectively, and the effects of YCHT on the two liver injury models were evaluated by analyzing the serum markers. YCHT showed significant effects on preventing liver injury progression induced by CCl_4_, and the closest or most suitable animal model for damp-heat jaundice syndrome may be the one induced by CCl_4_. To predict the potential molecular target proteins of YCHT by computer systems biology approaches [13]. Four and eight proteins were found to, respectively, associate with *Artemisiae scopariae* herba and Radix et Rhizome Rhei. Six components including rhein, emodin, 6, 7-dimethoxycoumarin not only directly interacted with target proteins which were proved by experiments, but also interacted with other related proteins. Eight components such as isofradin-3-O-glycoside could only play assistant roles by interacting with related proteins. It is expected that it would be helpful for understanding the molecular mechanism of YCHT. Taken together, the protocol developed in the study may lead to a deeper understanding of a system as a whole in the mechanism of YCHT.

Proteomics techniques have become key tools in the development of systems biology, and the use of proteomics techniques is crucial for the understanding and interpretation of TCM [14]. Integration of proteomics efforts with TCM has driven the development of a new drug discovery paradigm. Previous report showed that proteomics approaches had been used to study hepatic and serum protein expression changes in bile duct ligated rats following YCHT treatment for 27 days [15]. Interestingly, YCHT treatment caused a statistically significant downregulation in the secretion of monocyte chemoattractant protein-1 and tissue inhibitor of metalloproteinase-1 in bile duct ligated rats with fibrosis. It suggested that the therapeutic effects of YCHT on liver diseases might be associated with its lipid biosynthesis regulation. To identify the possible target proteins of YCHT, proteomics was performed and proteins altered after YCHT treatment were identified by MALDI-TOF/TOF-MS [16]. Interestingly, 15 modulated proteins were identified, out of which 7 were found to be significantly altered by YCHT. YCHT treatment caused a statistically significant downregulation of zinc finger protein 407, haptoglobin, macroglobulin, and alpha-1-antitrypsin; significant upregulation of transthyretin, vitamin D-binding protein, and prothrombin which appear to be involved in metabolism, energy generation, chaperone, antioxidation, signal transduction, protein folding, and apoptosis. It sheds light on the therapeutic mechanism of YCHT from a molecular perspective. Of note, the possible contributions of these proteins to the action of YCHT provide potential opportunities for the development of liver therapeutics. 

## 3. Quality Control of YCHT Samples 

Following the original description in “*Shanghan Lun*,” an ancient traditional Chinese medical text, YCHT was prepared in the following procedure. *Flos Artemisiae* (18 g) was decocted to a boil with 1400 mL of distilled water, and the boil was maintained until 700 mL of water remained. Then 9 g *Gardeniae Jasminoidis*, *Fructus* and 6 g Radix et Rhizoma *Rhei* were added, and kept boiling for 10 min. The solution was filtered through 5 layer gauzes and manufactured to a concentration of 1 g crude drug·mL^−1^ (this concentration solution was used for oral administration), and finally freeze-dried to powder for experiments. A vast majority of TCM are traditionally administered as individually prepared water decoction (tang) which is rather complicated in practice and that hampers straight production of more convenient application forms. Modernize technology may overcome these difficulties to traditional decoctions, and their quality can often not solely be attributed to the single marker compounds.

**Figure 2:**
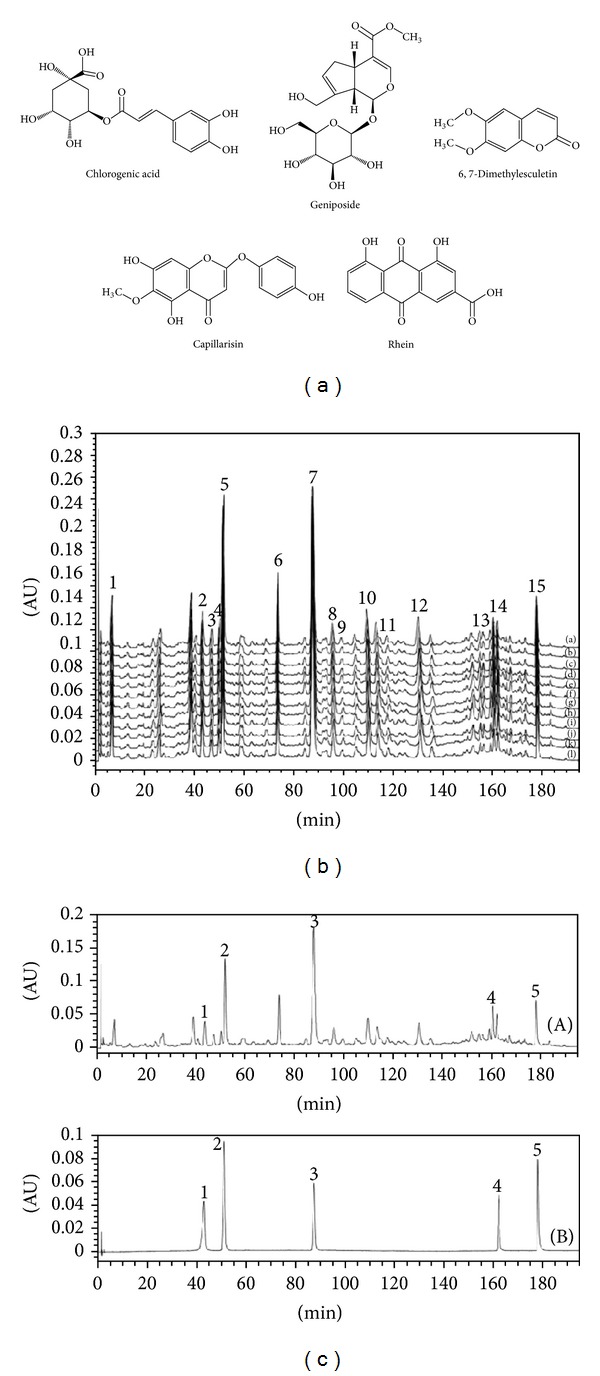
Chemical structure of chlorogenic acid, geniposide, 6,7-dimethylesculetin, capillarisin, and rhein. (b) Comparison of fingerprint profiles of twelve batches of YCHTE samples. Among fifteen representative general peaks, the peaks 2, 3, 6, 7, 8, 9, 10, 11, 12, and 14 originated from the flowers of *Artemisia capillaries* Thunb, the peaks 2, 4, and 5 originated from the fruits of *Gardenia jasminoides* Ellis, and the peaks 1, 13, and 15 originated from the roots of *Rheum officinale* Baill. The peaks 2, 5, 7, 14, and 15 are of chlorogenic acid, geniposide, 6,7-dimethylesculetin, capillarisin, and rhein, respectively. The fingerprint chromatograms of twelve batches of YCHT samples which are consisted of herbal drugs collected from different places of production, is from chromatogram a to chromatogram j. (c) HPLC chromatogram of (a), YCHTE in methanol; (b), the chemical references of five analytes in methanol: (1) chlorogenic acid; (2) geniposide; (3) 6,7-dimethylesculetin; (4) capillarisin; and (5) rhein.

A completely validated method based on HPLC coupled with photodiode array detector (HPLC-UV) was used for evaluating and controlling quality of YCHT [17]. First, HPLC-UV fingerprint chromatogram of YCHT was established for preliminarily elucidating amount and chromatographic trajectory of chemical constituents in YCHT. Second, for the first time, five mainly bioactive constituents (Figure 2(a)) in YCHT were simultaneously determined based on fingerprint chromatogram for a further controlling of the quality of YCHT quantitatively. The developed method that was applied to analyze 12 batches of YCHT samples which consisted of herbal drugs from different places of production, showed acceptable linearity, intraday, interday precision, and accuracy (Figure 2(b)). As a result, fingerprint chromatogram determined 15 representative general fingerprint peaks (Figure 2(c)). The contents of five analytes in different batches of YCHT samples do not indicate significant difference. So, the developed HPLC-UV method is a more fully validated and complete method for evaluating and controlling the quality of YCHT.

## 4. Compounds Derived from YCHT

TCM has been a rich source of lead drug discovery, based on its capability to create unique and diverse chemical structures. *Artemisinin* (Qinghaosu), for example, is an extract that is prepared from the *Artemisia annua*. It is now a very promising antimalarial drug. Efficient detection and rapid characterisation of natural products play key roles as an analytical support in the work of natural products chemists. In order to perform an efficient screening of TCM, the recent introduction of the optical UPLC coupled to MS represents a powerful complement to the LC-MS screening. The use of the hyphenated techniques allows the rapid structural determination of plant constituents with only a few minutes. With such an approach, the time-consuming isolation of common natural products is avoided and an efficient targeted isolation of compounds presenting biological features can be performed.

Chemical studies on the composition herbs *Flos Artemisiae, Gardeniae Jasminoidis, Fructus *andRadix et Rhizoma Rhei, have been conducted, respectively. Constituent compounds such as coumarin, flavone, chromone, anthraquinone, organic acids, enyne, triterpene, and sterides have been experimentally verified. Based on the chromatograms of rat plasma after oral administration of YCHT, UPLC-MS method was established in order to identify the effective constituents *in vivo* [18]. The fingerprints of the samples were established, with 45 compounds in YCHT and 21 compounds in serum containing drug were identified by comparing their retention time and ESI-MS data with those obtained from reference compounds. Two of the 21 *in vivo* compounds were metabolites and others were of the original form of the compounds *in vitro*. The constituents in rat plasma after oral administration of YCHT were well separated and identified by using their retention time and mass spectra. It is suitable for identifying the constituents in serum after oral administration of YCHT and provides helpful chemical information for further pharmacology and active mechanism research on the medical formula.

## 5. Pharmacokinetic Studies

As TCM is attractive as a pool of chemical compounds and natural products for medicinal use, increasing attention is being paid to the scientific evaluation of TCM. Pharmacokinetic study on active constituents in YCHT is a good way to predict a variety of events related to its efficacy. Considering the growing significance of a potential beneficial role for human health, detailed *in vivo* pharmacokinetic studies of TCM by proper administration route such as oral administration are required. To explore the bioactive components in YCHT, it is necessary to further study *in vivo *pharmacokinetic characteristics of multiple absorbed components and find out the optical time-course behavior of new leads in drug discovery. In order to screen multiple constituents in YCHT, the comprehensive investigation on the kinetic profile is necessary. Therefore, a sensitive UPLC-MS method was first developed to screen the potentially bioactive components *in vivo *after a single oral administration of YCHT [19]. The developed method was successfully applied to monitor the pharmacokinetic time-course of 21 compounds, and they were grouped into 3 clusters using pattern recognition approaches. Comparing the body dynamics of each composition, D, G, and R was the initial choice of the compounds as the candidate components. It found that D was distributed and eliminated rapidly. Tissue distribution showed the highest level was observed in liver, followed by the kidney and spleen; the lower level appeared in the muscle, thyroid, and adrenal [20]. It was not detected in the brain which indicated that D does not cross the blood-brain barrier after oral administration. 

A combined HPLC method with SPE procedure for determination of D and G in rat plasma has also been successfully established [21]. The developed method showed acceptable linearity, precision, accuracy and stability, which has been applied to studies of the effect of formulae compatibility on pharmacokinetics. It demonstrated that formulae compatibility could significantly affect pharmacokinetics of D and G in rat plasma. With change of formulae composition, the pharmacokinetics of D and G showed obvious change; however, every compatible herbal drug exhibited different effect on pharmacokinetics of D and G, which all could contribute to special priority of compatible administration of YCHT. The pharmacological effect of TCM is an interaction result of a host of bioactive compounds. Continuing work was to elucidate drug-interaction property of major active components D, G, and R as marker compounds, based on the pharmacokinetic characteristics. DGR significantly intensified the therapeutic efficacy as indicated by the modern biochemical analysis. A simple, sensitive and reliable RP-HPLC-DAD method has been developed to simultaneously quantify the D, G, and R which are the active ingredients from YCHT, performing drug-interaction pharmacokinetics studies *in vivo* [22]. It successfully demonstrated that this method has excellent and satisfactory selectivity, sensitivity, linearity, precision, accuracy, and recovery. DGR combination could significantly increase the plasma level, slow elimination rate more than any one or two of the three individual compounds, which may be an indication of a synergism. These observations indicate that TCM usually takes multicomponents to yield their efficacies, and have better effects than a single drug alone, and it may provide a promising design in natural products and new combination medicine pattern. 

## 6. Systems Biology on Synergistic Effect

To gain insight into the complex biochemical mechanisms of the effective therapy, both metabolomics and proteomics were incorporated to analyze the effects of the active components of YCHT [8]. UPLC-MS, MALDI-TOF MS/MS, and 2-DE were employed to systematically analyze the synergism of DGR at the levels of the proteome and metabolome, exploring some of the key molecular mechanisms that underlie synergistic effects. The approach extends the well-established concept of combinatorial therapeutics and identifies new potential strands of investigation that involve multicomponent combinations that target multiple pathways. It showed that the combined* in vivo* use of the active components of YCHT exerts more profound therapeutic effects than any component used individually. The biochemical analysis and immunohistochemical assay further support the cooperation of D and G with R in upregulating BCL-2 and downregulating FAS, with the strongest effect occurring with the DGR combination, thereby supporting the rationale of using YCHT for treating HI. It provides mechanistic insight into the synergistic effect. Metabolomic trajectory analysis indicated that each dosing group could be regulated back to baseline levels of the control group, but the maximum synergic effect was observed in rats treated with the DGR combination as opposed to mono- or bitherapies. These observations support the rationale of this formula wherein the compounds mutually reinforce each other. It found that DGR activated an array of factors that are involved in energy, amino acid, nucleotide, fatty acid, and cofactor and vitamin metabolism. It is important to note that based on our results, DGR targets not only immunity and metabolism but also key regulatory pathways that are used in transport, signal transduction, and cell growth and proliferation, thereby helping to restore the normal function.

## 7. Conclusion

Current tide may be turning back to nature in the search for new drug candidates [23–25]. Shifting the current drug discovery paradigm from “finding new drugs” to “screening natural agents” may be helpful for overcoming the “more investment, fewer drugs” challenge. One advantage of TCM therapeutics is the “synergism,” that is, the combinational effects to be greater than that of the individual drug. Making active component therapeutics a systematic approach offers new treatment opportunities. There has been a significant increase in the clinical use of combinatorial intervention in YCHT to achieve synergistic interactions that are capable of producing a sufficient effect at low doses. However, finding ways to research the synergistic combinations from numerous pharmacological components is still an ongoing challenge. Currently, systems biology approach is in line with the holistic concept and practices of TCM and will help to understand the molecular mechanisms of hepatoprotective effects of YCHT as a case study. We hope these studies will lay the foundation for dissecting the synergistic action of multicomponents from TCM and offer bright prospects for the control of complex diseases. 

## References

[1] Drews J (2000). Drug discovery: a historical perspective. *Science*.

[2] Zhang L, Ren X, Alt E (2010). Chemoprevention of colorectal cancer by targeting APC-deficient cells for apoptosis. *Nature*.

[3] Perelson AS, Essunger P, Cao Y (1997). Decay characteristics of HIV-1-infected compartments during combination therapy. *Nature*.

[4] Zhang Q, Mao J, Liu P (2009). A systems biology understanding of the synergistic effects of arsenic sulfide and Imatinib in BCR/ABL-associated leukemia. *Proceedings of the National Academy of Sciences of the United States of America*.

[5] Gao M, Nettles RE, Belema M (2010). Chemical genetics strategy identifies an HCV NS5A inhibitor with a potent clinical effect. *Nature*.

[6] Zheng P, Wang K, Zhang Q (2005). Systems analysis of transcriptome and proteome in retinoic acid/arsenic trioxide-induced cell differentiation apoptosis of promyelocytic leukemia. *Proceedings of the National Academy of Sciences of the United States of America*.

[7] Wang L, Zhou G, Liu P (2008). Dissection of mechanisms of Chinese medicinal formula Realgar-Indigo naturalis as an effective treatment for promyelocytic leukemia. *Proceedings of the National Academy of Sciences of the United States of America*.

[8] Wang X, Zhang A, Wang P (2013). Metabolomics coupled with proteomics advancing drug discovery towards more agile development of targeted combination therapies. *Molecular and Cellular Proteomics*.

[9] Zhang A, Sun H, Yuan Y, Sun W, Jiao G, Wang X (2011). An in vivo analysis of the therapeutic and synergistic properties of Chinese medicinal formula Yin-Chen-Hao-Tang based on its active constituents. *Fitoterapia*.

[10] Ogasawara T, Morine Y, Ikemoto T, Imura S, Shimada M (2008). Beneficial effects of Kampo medicine Inchin-ko-to on liver function and regeneration after hepatectomy in rats. *Hepatology Research*.

[11] Wang X, Lv H, Sun H (2008). Metabolic urinary profiling of alcohol hepatotoxicity and intervention effects of Yin Chen Hao Tang in rats using ultra-performance liquid chromatography/electrospray ionization quadruple time-of-flight mass spectrometry. *Journal of Pharmaceutical and Biomedical Analysis*.

[12] Cao H, Sun H, Jiang X (2009). Comparative study on the protective effects of Yinchenhao Decoction against liver injury induced by *α*-naphthylisothiocyanate and carbon tetrachloride. *Chinese Journal of Integrative Medicine*.

[13] Liu T, Huang HB, Lin ZC, Liu Q, Zhu W (2011). Exploring the potential molecular target proteins of yinchenhao decoction using computer systemic biology. *Zhong Yao Cai*.

[14] Wang X, Zhang A, Sun H (2012). Future perspectives of Chinese medical formulae: chinmedomics as an effector. *OMICS*.

[15] Lee T, Chang H, Kuo J, Shen J (2009). Changes of hepatic proteome in bile duct ligated rats with hepatic fibrosis following treatment with Yin-Chen-Hao-Tang. *International Journal of Molecular Medicine*.

[16] Sun H, Zhang A, Yan G (2013). Proteomics study on the hepatoprotective effects of traditional Chinese medicine formulae Yin-Chen-Hao-Tang by a combination of two-dimensional polyacrylamide gel electrophoresis and matrix-assisted laser desorption/ionization-time of flight mass spectrometry. *Journal of Pharmaceutical and Biomedical Analysis*.

[17] Wang X, Lv H, Sun H (2008). Quality evaluation of Yin Chen Hao Tang extract based on fingerprint chromatogram and simultaneous determination of five bioactive constituents. *Journal of Separation Science*.

[18] Wang X, Sun W, Sun H (2008). Analysis of the constituents in the rat plasma after oral administration of Yin Chen Hao Tang by UPLC/Q-TOF-MS/MS. *Journal of Pharmaceutical and Biomedical Analysis*.

[19] Wang X, Sun H, Zhang A, Jiao G, Sun W, Yuan Y (2011). Pharmacokinetics screening for multi-components absorbed in the rat plasma after oral administration traditional Chinese medicine formula Yin-Chen-Hao-Tang by ultra performance liquid chromatography-electrospray ionization/quadrupole- time-of-flight mass spectrometry combined with pattern recognition methods. *Analyst*.

[20] Yin Q, Sun H, Zhang A, Wang X (2012). Pharmacokinetics and tissue distribution study of scoparone in rats by ultraperformance liquid-chromatography with tandem high-definition mass spectrometry. *Fitoterapia*.

[21] Lv H, Sun H, Sun W (2008). Pharmacokinetic studies of a Chinese triple herbal drug formula. *Phytomedicine*.

[22] Zhang A, Sun H, Wang X, Jiao G, Yuan Y, Sun W (2012). Simultaneous in vivo RP-HPLC-DAD quantification of multiple-component and drug-drug interaction by pharmacokinetics, using 6,7-dimethylesculetin, geniposide and rhein as examples. *Biomedical Chromatography*.

[23] Zhang A, Sun H, Wang X (2013). Recent advances in natural products from plants for treatment of liver diseases. *European Journal of Medicinal Chemistry*.

[24] van der Greef J, Martin S, Juhasz P (2007). The art and practice of systems biology in medicine: mapping patterns of relationships. *Journal of Proteome Research*.

[25] Archin NM, Liberty AL, Kashuba AD (2012). Administration of vorinostat disrupts HIV-1 latency in patients on antiretroviral therapy. *Nature*.

